# Assessment of Azathioprine-Associated Lymphopenia Incidence Rates in Polish Children with Inflammatory Bowel Disease and Autoimmune Hepatitis: A Retrospective Study

**DOI:** 10.3390/children12081093

**Published:** 2025-08-20

**Authors:** Katarzyna Bąk-Drabik, Anna Kaput, Anna Jarzumbek, Katarzyna Górowska-Kowolik, Agnieszka Szymlak, Agnieszka Krzywicka, Piotr Adamczyk, Jarosław Kwiecień

**Affiliations:** 1 Department of Paediatrics, Faculty of Medical Sciences in Zabrze, Medical University of Silesia, 40-055 Katowice, Poland; ajarzumbek@sum.edu.pl (A.J.); kkowolik@sum.edu.pl (K.G.-K.); agnieszka.szymlak@sum.edu.pl (A.S.); jkwiecien@sum.edu.pl (J.K.); 2 Faculty of Medical Sciences in Zabrze, Students Association, Medical University of Silesia, 40-055 Katowice, Poland; a.kaput@wp.pl; 3 Clinical Hospital No. 1, 41-800 Zabrze, Poland; akrzywicka@szpital.zabrze.pl; 4 Department of Paediatrics, Faculty of Medical Sciences in Katowice, Medical University of Silesia, 40-055 Katowice, Poland; padamczyk@sum.edu.pl

**Keywords:** azathioprine, child, lymphopenia, opportunistic infection, inflammatory bowel disease, autoimmune hepatitis

## Abstract

Background and objective: Thiopurines (azathioprine (AZA) and 6-mercaptopurine (6-MP)), used to maintain remission in inflammatory bowel diseases (Crohn’s disease (CD), ulcerative colitis (CU)) and autoimmune hepatitis (AIH), are responsible for a number of adverse effects. One is leukopenia, mainly due to neutropenia and less known lymphopenia. This study aimed to assess the incidence rate of lymphopenia in pediatric patients with CD, CU, and AIH treated with azathioprine (AZA) and to evaluate the impact of lymphopenia on the occurrence of opportunistic infections and its relationship with disease activity, treatment, and nutritional status. Materials and methods: A retrospective analysis was carried out in ninety-eight (98) paediatric patients, suffering from CD, CU, or AIH and treated with AZA, in order to assay blood cell count and thiopurine metabolite levels, assess the mean AZA dose, measure the anthropometric parameters, evaluate disease activity vs. the treatment administered, and to find out concomitant infections. Results: Lymphopenia was diagnosed in twenty-two (22) children and evaluated as severe in two (2) cases, which were associated with treatment discontinuation. The percentage of patients with lymphopenia in the CD group (34.5%) was significantly higher vs. the CU (3.7%) and AIH (7.7%) groups. The prevalence rates of the patients with low and moderate-to-high disease activity were 13.9% and 46.1%, respectively. The patients with lymphopenia demonstrated higher prevalence rates of mild respiratory tract and skin infections (identified in 32%). No cases of opportunistic infections were reported. Conclusions: Lymphopenia affected approximately one-quarter of the patients observed, the condition being transient in most cases and not demanding any therapy modifications. In no case was it associated with the occurrence of any opportunistic infections. It was significantly more common in the patients with Crohn’s disease and the subgroup with a more intense course of the disease, obviously suggesting a need for more frequent follow-up of the patients in those subgroups. The AZA therapy did not seem to be associated with lymphopenia occurrence in any significant way.

## 1. Introduction

Thiopurines (azathioprine and 6-mercaptopurine; AZA/6-MP) are commonly used to maintain remission in many autoimmune diseases. In addition, they are applied in haematology as a component of cancer chemotherapy and transplantology following organ transplantation. The history of their use dates back more than fifty years. The mechanism of their action involves the incorporation of purine thioanalogues into the DNA chain, with the consequent blocking of nucleic acid biosynthesis and the inhibition of immune cell proliferation. A full immunosuppressive effect is usually achieved after 14 weeks of treatment [1]. Azathioprine, as a pro-drug, undergoes biotransformation in the liver and the kidneys to 6-mercaptopurine, which readily enters the body’s cells and is metabolised to purine thioanalogues. The rate of this metabolism is individually variable and depends on multiple enzyme systems. Therapy optimisation is based on its clinical efficacy assessments, the stability of blood cell counts and periodic assays of thiopurine metabolites: 6-TGN and 6-methyl-mercaptopurine (6-MMP), in order to establish an individualised dosing regimen, thereby reducing the risk of drug toxicity [2,3,4]. Myelosuppression is one of the most serious side effects of thiopurines, being attributed to 6-thioguanine (6-TGN), one of the metabolites. Leukopenia is mainly the result of neutropenia. Following published reports, the incidence rate of myelotoxicity is estimated at 7% of the patients treated, mainly in the first few months of the treatment [2,5].

Much less is known about the prevalence of lymphopenia and its impact on the occurrence of opportunistic infections. Lymphopenia means a lymphocyte deficiency in peripheral blood, down to more than 1500 C/μL. Mild, moderate, severe, and very severe lymphopenia are defined by lymphocyte counts as 1499 and 1000 C/μL, 999–500 C/μL, 499–200 C/μL, and <200 C/μL, respectively [1]. There is a systematic increase in autoimmune diseases in paediatric patients, which translates into rising numbers of children treated with thiopurines. Data on lymphopenia, as secondary to the treatment in inflammatory bowel disease, depend on the definition criteria and the study methodology, indicating the range of 10–30% of patients treated with thiopurines [6,7]. However, severe infections have also been observed at lower prevalence rates of 4.6% and 5.4% in Crohn’s disease (CD) and ulcerative colitis (CU), respectively [8]. On the other hand, however, a cytotoxic effect, oriented at T lymphocytes, is somehow the target of the AZA/6-MP immunosuppressive therapy. Hence, some investigators tend to interpret mild to moderate lymphopenia as a parameter of sufficient immunosuppression and not solely as an adverse event. However, they recommend observation of the patients affected for the risk of an increased incidence of infection [1].

The research project was intended to provide evidence-based information on the prevalence rates of lymphopenia in a paediatric group of patients with CD, UC, and autoimmune hepatitis (AIH). Although lymphopenia is commonly identified in adult populations, it remains rather inadequately studied in paediatric patients. There is also a striking scarcity of information on the processes that may enhance the prolymphocytic effect of azathioprine, such as malnutrition, the type and severity of an underlying disease, or a concomitant pharmacotherapy. Little is also known of the risk of severe opportunistic infections resulting from azathioprine-induced lymphopenia in children, which, for many clinicians, could provide some important information for effective care planning.

To the authors’ knowledge, there are no studies that assess the incidence of azathioprine-associated lymphopenia in the Polish children’s population. Polymorphisms in genes encoding enzymes of the azathioprine metabolic pathway affect the risk of adverse effects, including myelosuppression. This results in population differences. Therefore, this work is unique and makes a valuable contribution to understanding the safety of this drug, identifying risk groups that require close monitoring.

This study’s primary goal was to assess the incidence rate of lymphopenia in paediatric patients with CD, CU, and AIH treated with AZA, while the secondary goals included an evaluation of the impact of the lymphopenia on the occurrence of opportunistic infection and its relationship with disease activity, treatment, and nutritional status, as well.

## 2. Materials and Methods

### 2.1. Patients

We conducted a retrospective analysis of the medical records of 121 patients with CD, CU, and AIH, all of them being treated at a tertiary paediatric gastroenterology centre in Poland between January 2017 and December 2023, with fixed-dose AZA to maintain remission for at least 3 months.

The medical records of those patients were screened during the therapy for lymphopenia (defined as <1500 lymphocytes/μL). The patients with insufficient documentation of either lymphocyte counts or disease activity, treatment duration, 6-TGN concentration, or concomitant medication(s) were excluded from the analysis. Eventually, a complete analysis was carried out in a group of ninety-eight (98) patients. In that group, fifty-eight (58) patients were diagnosed with Crohn’s disease, twenty-seven (27) with ulcerative colitis, and thirteen (13) with autoimmune hepatitis. All the IBD patients were treated according to the European Crohn’s and Colitis Organisation (ECCO) guidelines, while the AIH patients were treated according to the European Society for Paediatric Gastroenterology Hepatology and Nutrition (ESPEGHAN) guidelines. Disease activity was assessed using PUCAI (the Paediatric Ulcerative Colitis Activity Index) and PCDAI (the Paediatric Crohn’s Disease Activity Index). Remission in the patients with IBD was defined as a score below 10 on the PUCAI and PCDAI scales.

Biochemical remission in AIH was assessed as the normalisation of liver test activity and IgG [9].

The criteria for inclusion into the study entailed the diagnosis of either IBD or AIH in accordance with the current guidelines (ESPEGHAN and/or ECCO), lymphocyte counts within the normal range before AZA treatment, AZA treatment duration for at least 3 months, and a patient’s age of ≥ 4 years. The exclusion criteria comprised missing data in patient medical records, concomitant chronic diseases, an age of <4 years, or a history of lymphopenia.

The dosage of azathioprine depended on 6-TGN concentration. Dose modification was carried out repeatedly until the optimum concentration of 6-TGN was achieved (230–450 pmol/8 × 10^8^ erythrocytes and 6-MMP below 5700 pmol/8 × 10^8^ erythrocytes).

The following parameters were analysed: gender, the type of disease, the date of lymphopenia appearance, the severity of lymphopenia, the patient’s age when lymphopenia occurred, the age of azathioprine inclusion, the time to the onset of lymphopenia, the type of treatment administered, disease activity, anthropometric parameters, and 6-TGN levels in erythrocytes.

### 2.2. Methods

A retrospective analysis of blood counts was performed in each patient at the time of diagnosis, then at the time of AZA inclusion, and at each patient’s follow-up at the Gastroenterology Department. Anthropometric parameters, disease activity, and the treatment applied were evaluated at the time of AZA inclusion and at the onset of lymphopenia. Information on the infection was obtained from the patient’s medical history, and the diagnosis was obtained by an outpatient physician on the basis of clinical symptoms and without bacteriological confirmation. The occurrence of more than six infection episodes per year was considered frequent. A mild infection was defined as a condition without fever, treated symptomatically, without antibiotic therapy or a need for inpatient care. The infections with *Candida albicans*, *Pneumocystis jiorvecii*, *Apsergillus*, Human Immunodeficiency Virus (HIV), *Cytomegalovirus* (CMV), *Herpes Simplex Virus* (HSV), or *Mycobacterium tuberculosis* were considered opportunistic infections. Lymphopenia persisting for more than 3 months was considered a persistent condition. The white blood cells, including lymphocytes, were assayed by fluorescence flow cytometry, using a semiconductor laser in a Sysmex analyser series XN-L. AZA metabolite (6-TGN) levels were assayed at an outsourced analytical laboratory. In summary, the cells, isolated from venous EDTA (*ethylenediaminetetraacetic acid*) blood samples, were washed with an isotonic buffer three times and then lysed using thermal disruption. Subsequently, the lysates were deproteinised by incubation in acidic conditions and centrifuged for at least 15 min at >10,000 rcf to remove cellular debris. The cleared lysates were analysed by high-performance liquid chromatography (HPLC) against a reversed phase (RP) and by detection at 300–350 nm (using a UV-VIS detector). The obtained concentrations were quantified using the AUC (area under the curve) method, comparing the values against a standard curve obtained with synthetic calibrators of known concentrations. Such raw readouts were normalised based on each sample’s RBC (red blood cell count). The final results were calculated as pmol/8 × 10. The study was conducted in accordance with the Declaration of Helsinki and approved by the Ethics Committee of the Medical University of Silesia, as confirmed in a dedicated protocol. All the data, routinely collected at our Pediatric Centre, were anonymised in line with applicable procedures.

### 2.3. Statistical Analysis

A statistical analysis was performed using the Statistica 12 software (StatSoft, Tulsa, OK, USA). The mean values and standard deviations were used for descriptive statistics of continuous variables. Absolute and percentage values were provided for qualitative variables. A Student’s *t*-test for independent samples or the Mann–Whitney U test was applied for the comparative analyses of continuous variables, depending on data distribution, which was verified by the Shapiro–Wilk test. Comparisons of qualitative feature rates were performed by the chi-square test. Logistic regression with lymphopenia as the dependent variable and potential explanatory variables, including sex, age at diagnosis, diagnosis of Crohn’s disease, 6-TGN serum concentration, disease activity, and BMI-SDS, was used for multivariate analysis. The statistical significance of results was assumed at *p* < 0.05 for all the statistical analyses carried out.

## 3. Results

### 3.1. The Prevalence Rate of Lymphopenia in the Study Group

Lymphopenia was found in twenty-two (22) children (22.4%). The full clinical characteristics of the whole study group and subgroups with lymphopenia and without lymphopenia are presented in Table 1.

**Figure 1:**
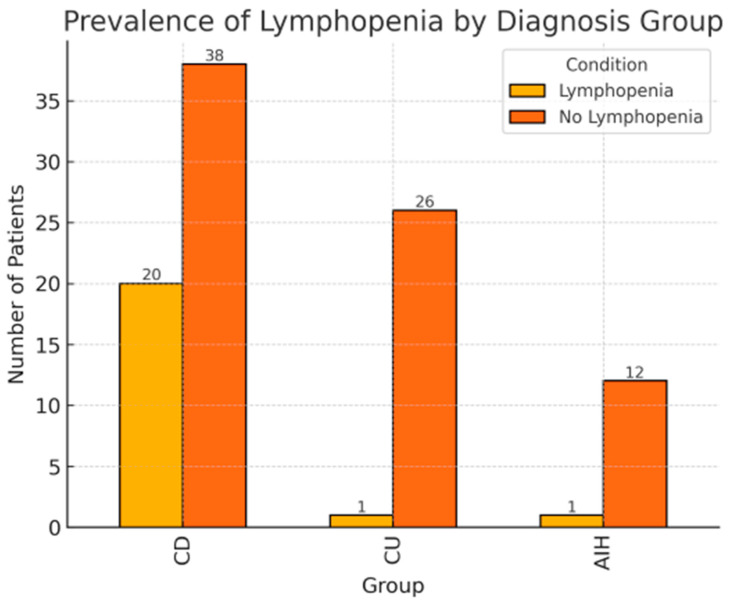
Profiles of the patients with and without lymphopenia depending on underlying disease.

The proportion of patients with lymphopenia in the CD group (34.5%) was significantly higher when compared to the CU (3.7%) and AIH (7.7%) groups (chi-square test = 11.9, *p* < 0.01) (Figure 1). Among the patients with diagnosed lymphopenia, 20 children (91%) demonstrated a moderate (999–500 C/μL) and 2 children (9%) a severe (499–200 C/μL) level. Severe lymphopenia resolved in either case after AZA discontinuation. In only four (4) (18%) cases was lymphopenia persistent, i.e., lasting throughout the patient’s follow-up. In the remaining cases, lymphopenia was a transient incident, lasting not longer than three (3) months; neither was it associated with treatment modification. The mean time from the AZA inclusion to lymphopenia occurrence was 14.3 ± 11.7 months.

### 3.2. Differences Between IBD (CU, CD) and AIH Patients with Lymphopenia and the No-Lymphopenia Subjects

**Figure 2:**
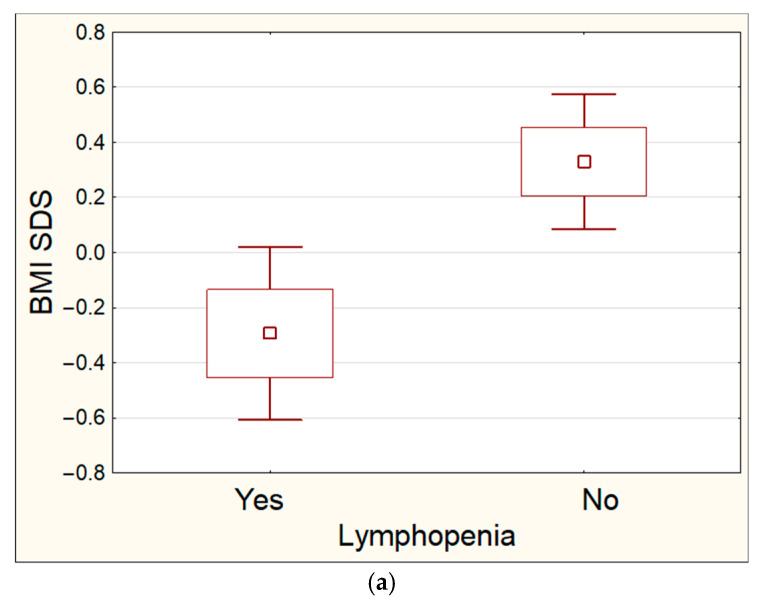

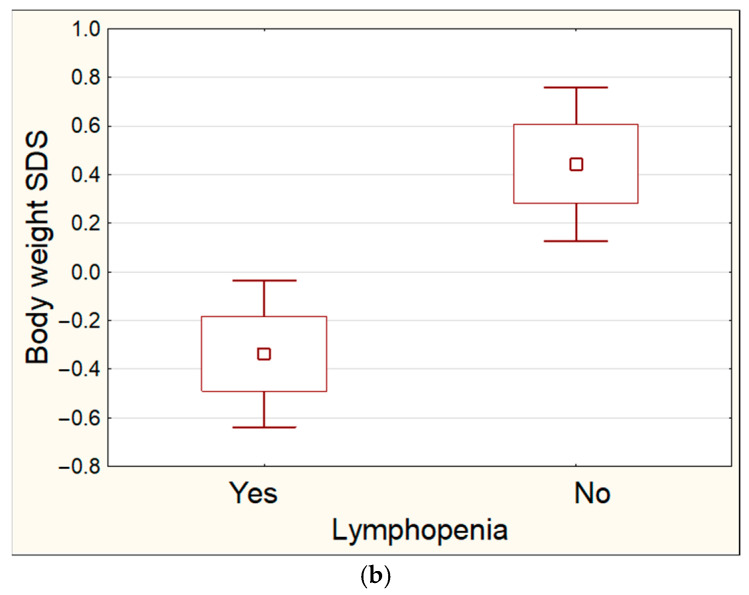
(**a**) Comparison of the BMI SDS f the patients with and without diagnosed lymphopenia (*p* < 0.05). (**b**) Comparison of the Body Weight SDS of the patients with and without diagnosed lymphopenia (*p* < 0.05).

The mean BMI-SDS also differed significantly between the patients with and without lymphopenia (−0.29 ± 0.75 vs. 0.33 ± 1.09) (*p* < 0.05) (Figure 2a).

The mean weight SDS differed significantly between the patients with lymphopenia and those without. (−0.33 ± 0.72 vs. 0.44 ± 1.41) (*p* < 0.05) (Figure 2b).

### 3.3. The Prevalence of Lymphopenia in Relation to Disease Activity

The percent rate of the patients with lymphopenia in the subgroup with low disease activity was 13.9%, which was statistically significantly lower when compared to the subgroup with either moderate or high disease activity, where it was 46.1% (chi-square test = 11.42, *p* < 0.001).

### 3.4. Associations Between Lymphopenia, AZA Treatment, Disease Duration, AZA Onset Time Point, AZA Dose, Blood Cell 6-TGN Concentration, Additional Treatment, and the Patient’s Gender

No significant differences were observed among the appearance of lymphopenia and the AZA treatment duration, the time of diagnosis, the time of AZA onset, the 6-TGN concentrations, the concomitant therapy, and the patient’s gender.

### 3.5. Comparison of the Patients with and Without Lymphopenia in Terms of Infection Occurrence

No cases of opportunistic infections were reported. The patients with lymphopenia demonstrated higher prevalence rates of mild respiratory tract and skin infections compared to subjects without lymphopenia (32% vs. 0%, respectively; Chi-square test = 26.04; *p* < 0.001).

### 3.6. Logistic Regression Analysis

Finally, the variables that showed potentially significant associations with the occurrence of lymphopenia in univariate analyses were included as independent variables in the logistic regression analysis. This analysis showed lymphopenia occurrence is statistically significantly associated with the diagnosis of CD (Odds ratio 10.6; 95%CI 2.0–56.8) and with high disease activity (Odds ratio 6.2; 95%CI 1.8–21.8). Associations with the remaining parameters considered (sex, age at disease onset, 6-TGN concentration, and BMI-SDS) turned out to be statistically insignificant. Detailed results are presented in Table 2.

## 4. Discussion

Azathioprine (AZA) is one of the oldest known immunosuppressive drugs. AZA therapy exerts specific effects on lymphocyte populations [10]. One of its effects is pro-inflammatory T-cell action suppression [11]. In general, AZA therapy affects T cells to reduce their activation. T-lymphocyte activation requires two elements: a TCR receptor ligand and a costimulatory signal. The absence of the latter, as a result of the AZA blocking the Rac-1 molecule activation, inhibits the response of T-lymphocytes to the antigen and promotes the process of their apoptosis [10]. Adequate to the drug action mechanism, and one of the phenomena observed in AZA-treated patients is the varying degree of lymphopenia. Current studies focus on the incidence rate and possible consequences of this observation, trying to identify possible risk factors [6,12].

The factors responsible for the risk of lymphopenia during AZA therapy are not unambiguous, while polytherapy is sometimes assumed to be one of such factors. The patients on concomitant steroid therapy were described as being at risk of lymphopenia [6,12,13]. In our study, no association was identified between polypragmasy and lymphopenia. However, some interactions between AZA and 5-ASA are a known phenomenon, affecting treatment efficacy and bringing a risk of potential side effects. A synergistic effect of both drugs seems likely, due to 5-ASA inhibition of thiopurine methyltransferase (TPMT) activity, which results in increased serum 6-TG levels and may be responsible for the myelosuppressive effect [13]. Nguyen et al. evaluated seventy-one (71) children with IBD treated for at least one year with AZA. Lymphopenia was diagnosed more frequently in the patients treated with AZA in combination with the aminosalicylate therapy (5-ASA). Increased serum thiopurine metabolites (6-TGN) were also found in those subjects [12].

The risk of leukopenia is increased in patients with high glutathione-S-transferase (GST) activity and low TPMT activity. The patients with low TPMT activity, as a result of the deposition of AZA metabolites in their bone marrow, represent a risk group for severe neutropenia. On the other hand, leukopenia appears to be more of a GST-M1 mutation of the gene for GST [7,14]. It should be noted that, regarding the patients with a different activity of the above-mentioned enzymes, according to the accepted principles of AZA metabolism, an increased concentration of 6-TGN should be observed, and the amount of metabolites formed should be in relation to the dose of the drug. In addition, a relationship has been reported between the dose of the drug and the concentrations of its metabolites and their effect on leukocyte and lymphocyte counts [14,15,16].

Our study found no correlation between the drug dose, 6-TGN concentration, and lymphopenia prevalence rates. Similarly to our findings, a meta-analysis performed in 2020 on, among other things, the toxicity of thiopurines, found no statistically significant correlation between the serum concentration of their metabolites and the concentration of lymphocytes in the peripheral blood of the patients in whom thiopurines were combined with other drugs. Regarding the patients remaining on monotherapy, this association has been described as weak [15]. This is supported by the results of other studies suggesting that the activity of enzymes involved in AZA metabolism is not the only risk factor for myelotoxicity [14,17].

In our study, no significant differences were observed among the appearance of lymphopenia and the AZA treatment duration, the time of diagnosis, the time of AZA onset, and the patient’s gender. In most of our patients, lymphopenia was a transient incident, and it was not associated with treatment modification. Other authors have reported similar observations. Al Rifai et al. followed up a group of fifty-two (52) adult patients who had started AZA therapy. Nearly 35% of the patients had developed lymphopenia, which was severe in more than half of them. In the majority of the patients, lymphopenia resolved spontaneously, not causing any severe infectious complications [6]. No increased incidence of lymphopenia was observed with regard to either age, gender, or IBD type (CD vs. CU) [6].

In our results, however, lymphopenia was significantly more frequent in the patients with Crohn’s disease (CD). We also found a difference between the prevalence of lymphopenia in patients with different disease activity.

Monasterio et al. described the case of a young patient with CD who had developed profound lymphopenia after initiation of a combined therapy with AZA and steroids. A withdrawal of AZA resulted in only a slight increase in CD4+ lymphocytes. A real improvement was only achieved after remission of inflammatory bowel disease. The authors of that article suggested that lymphopenia in IBD could be related not only to the therapy applied, but also to the high activity of the inflammatory bowel disease [18]. Detailed observations on the cause of lymphopenia in children with the so-called severe disease, including severe generalised inflammatory reactions, may be helpful to throw some light on this phenomenon. In these patients, several specific mechanisms responsible for the occurrence of lymphopenia have been described, some of which relate to the promotion of granulocytopoiesis at the expense of lymphopoiesis, while others control the inhibitory effect of neutrophils themselves on CD4+ lymphocyte proliferation and activity [19]. Although the observed group of our patients showed concomitance of lymphopenia and severe inflammation, taking into account the observational design of the study, it cannot be unequivocally suggested that an active, inflammatory process was, in addition to the AZA therapy, an independent, causative factor for lymphopenia. It is also worth noting that a high inflammatory disease activity, especially in CD patients, favours both malabsorption and weight loss. This may explain the lower BMI values observed in the group of children with lymphopenia. An increased risk of infectious diseases, including opportunistic and severe infections, appears to be one of the most serious possible consequences of lymphopenia.

There are scarce literature reports on possible sequelae, including infections, secondary to the AZA-induced lymphopenia. The lack of data, in particular, concerns paediatric patients exposed to an increased incidence of infections.

A concurrent use of other immunosuppressive drugs appears to be an additional risk factor. This is especially true for the AZA therapy combined with glucocorticosteroids and anti-TNF drugs [13,20]. According to the available data, most infectious complications involve the respiratory tract, the gastrointestinal tract, and the skin [2,13]. In a study evaluating the safety of AZA and 6-MP among children, 54% of the subjects showed good treatment tolerance. Only eight (8) of the ninety-five (95) patients suffered from recurrent infections, two of whom developed septicaemia. Three patients required discontinuation of the therapy due to infectious complications [21].

Opportunistic infections pose the greatest risk. The majority of available reports indicate an increased risk of severe viral infections (HSV, VZV, EBV, CMV) in patients treated with thiopurines [22,23,24]. In a study on adverse reactions during IBD pharmacotherapy in children, isolated cases of symptomatic EBV infection were reported in patients treated with AZA [22]. Cases of reactivation of latent HSV infection as a result of the suppressive effect of AZA on NK cell counts have also been reported [23]. Another study, conducted in a large group of patients treated with thiopurines, showed a nearly twofold higher risk of *P. jirovecii* infection in the paediatric population, compared to adults undergoing the same therapy [25]. However, none of the cited studies established a link between the occurrence of infection and AZA-induced myelosuppression/lymphopenia. In a study involving a group of 100 IBD patients treated with thiopurines, nine (9) cases of opportunistic infections, out of one hundred and sixty-one (161) episodes of lymphopenia were described. Only two of those involved severe lymphopenia. One opportunistic infection was reported in the control group. All the observed infections presented a mild course. No association was observed between the severity of leukopenia and the occurrence of infectious disease [1]. Our results also confirm the safety of AZA therapy, also in combination with other immunosuppressive and anti-inflammatory drugs.

We acknowledge the limitations of this study, including its observational retrospective design. The study attempted to approach the levels of thiopurine metabolites (6-TGN) as a potential risk factor for lymphopenia. However, the occurrence of thiopurine metabolism polymorphism was not taken into account, because in our center, we do not examine polymorphism in genes encoding enzymes, due to some economic conditions. Further studies are then needed to assess the relationship between lymphopenia and GST and TPMT activity. Considering our observations, it also seems important to determine the relationships among body weight, inflammatory disease activity, and the risk of drug-induced lymphopenia.

## 5. Conclusions

Lymphopenia was observed in approximately one-quarter of the patients in the studied group, primarily as a transient condition that did not require any modifications to their therapy in most cases. Notably, it was not associated with the development of opportunistic infections. The occurrence of lymphopenia was significantly more prevalent among patients with Crohn’s disease, and in the subgroup with higher disease activity, suggesting the necessity for more frequent monitoring of patients within these subgroups, even though AZA therapy itself was not significantly linked to the incidence of lymphopenia.

It is crucial to interpret these results with caution, considering the inherent limitations of the retrospective observational design, such as potential selection bias, a limited sample size, and the absence of long-term follow-up data. Therefore, further prospective studies are warranted.

## Figures and Tables

**Table 1:** Basic clinical features of the study group and characteristics of therapy.

Characteristic Feature	All Values	Lymphopenia	No Lymphopenia	*p*-Value
Total number of patients	98	22 (22.4%)	76 (77.5%)	
Females (percent)	47 (48%)	8 (36.4%)	39 (51.3%)	0.21
Males (percent)	51	14 (63.6%)	37 (48.7%)	
The age at diagnosis (years ± SD)	12.6 ± 3.2	13.3 ± 2.2	12.5 ± 3.4	0.31
The age at azathioprine therapy onset	13.6 ± 2.4	13.7 ± 2.2	13.6 ± 2.5	0.95
Crohn’s disease (CD)	58 (59%)	20 (90%)	38 (50%)	<0.01
Ulcerative colitis (UC)	27 (27%)	1 (4.5%)	26 (34%)	
Autoimmune hepatitis (AIH)	13 (13.3%)	1 (4.5%)	12 (15.8%)	
Infections	7 (7.1%)	7 (31.8%)	0	<0.001
Body weight SDS (mean ± SD)	0.27 ± 1.32	−0.33 ± 0.72	0.44 ± 1.41	<0.05
Body height SDS (mean ± SD)	0.22 ± 1.52	−0.20 ± 0.87	0.34 ± 1.65	0.14
BMI SDS (mean ± SD)	0.19 ± 1.05	−0.29 ± 0.75	0.33 ± 1.09	<0.05
6-TGN (median; interquartile range)	417.2; 218.0–562.6	312.8; 235.3–504.5	425.7; 214.5–573.6	0.29
Disease activity (the number of patients)				
Remission/mild	72	10	62	<0.001
moderate/severe	26	12	14	
Treatment type:				
Azathioprine	6	0	6	
Azathioprine + Aminosalicylates	40	6	34	
Azathioprine + Aminasalicylates + Steroids	32	6	26	
Azathioprine + Aminasalicylates + Biological therapy	20	10	10	
Daily dose of azathioprine	56.6 ± 20.9	53.9 ± 21.5	57.4 ± 20.7	0.77

**Table 2:** Logistic regression results with lymphopenia occurrence as the dependent variable. R2 = 0.27.

Variable	OR (95% CI)	SE	*p*-Value
Male sex	1.89 (0.57–6.26)	1.16	0.298
Age at diagnosis	1.01 (0.83–1.23)	0.10	0.904
Diagnosis of Crohn’s disease	10.61 (2.00–56.82)	9.08	**0.006**
6-TG concentration	1.00 (1.00–1.00)	0.00	0.456
High disease activity	6.21 (1.80–21.80)	3.98	**0.004**
BMI-SDS	0.70 (0.36–1.36)	0.24	0.294

OR: Odds Ratio; CI: Confidence Interval; SE: Standard Error; 6-TG: 6-thioguanine; BMI-SDS: Body Mass Index—Standard Deviation Score. Statistically significant *p*-values (<0.05) are in bold.

## Data Availability

The original contributions presented in the study are included in the article; further inquiries can be directed to the corresponding author: katarzyna.drabik@sum.edu.pl.
